# Plants of Genus *Mentha*: From Farm to Food Factory

**DOI:** 10.3390/plants7030070

**Published:** 2018-09-04

**Authors:** Bahare Salehi, Zorica Stojanović-Radić, Jelena Matejić, Farukh Sharopov, Hubert Antolak, Dorota Kręgiel, Surjit Sen, Mehdi Sharifi-Rad, Krishnendu Acharya, Razieh Sharifi-Rad, Miquel Martorell, Antoni Sureda, Natália Martins, Javad Sharifi-Rad

**Affiliations:** 1 Medical Ethics and Law Research Center, Shahid Beheshti University of Medical Sciences, Tehran 1983963113, Iran; bahar.salehi007@gmail.com; 2 Student Research Committee, Shahid Beheshti University of Medical Sciences, Tehran 1983963113, Iran; 3 Department of Biology and Ecology, Faculty of Sciences and Mathematics, University of Niš, Višegradska 33, 18000 Niš, Serbia; zstojanovicradic@yahoo.com; 4 Department of Pharmacy, Faculty of Medicine, University of Niš, Boulevard Dr Zorana Đinđića 81, 18000 Niš, Serbia; jekamatejic@gmail.com; 5 Department of Pharmaceutical Technology, Avicenna Tajik State Medical University, Rudaki 139, Dushanbe 734003, Tajikistan; shfarukh@mail.ru; 6 Institute of Fermentation Technology and Microbiology, Lodz University of Technology, 90-924 Łódź, Poland; hubert.antolak@gmail.com (H.A.); dorota.kregiel@p.lodz.pl (D.K.); 7 Molecular and Applied Mycology and Plant Pathology Laboratory, Department of Botany, Centre of Advanced Study, University of Calcutta, 35, Ballygunge Circular Road, Kolkata 700019, India; surjitsen09@gmail.com (S.S.); krish_paper@yahoo.com (K.A.); 8 Department of Medical Parasitology, Zabol University of Medical Sciences, Zabol 61663335, Iran; 9 Zabol Medicinal Plants Research Center, Zabol University of Medical Sciences, Zabol 61615585, Iran; razieh.sharifirad@gmail.com; 10 Department of Nutrition and Dietetics, Faculty of Pharmacy, University of Concepcion, Concepcion, 4070386 VIII-Bio Bio Region, Chile; 11 Research Group on Community Nutrition and Oxidative Stress and CIBEROBN (Physiopathology of Obesity and Nutrition), University of Balearic Islands, 07122 Palma de Mallorca, Spain; tosugo@hotmail.com; 12 Faculty of Medicine, University of Porto, Alameda Prof. Hernâni Monteiro, 4200-319 Porto, Portugal; 13 Institute for Research and Innovation in Health (i3S), University of Porto, 4200-135 Porto, Portugal; 14 Phytochemistry Research Center, Shahid Beheshti University of Medical Sciences, Tehran 11369, Iran; 15 Department of Chemistry, Richardson College for the Environmental Science Complex, The University of Winnipeg, Winnipeg, MB R3B 2E9, Canada

**Keywords:** *Mentha* genus, essential oil, chemotypes, plant extracts, culture conditions, food preservatives, antimicrobials

## Abstract

Genus *Mentha*, a member of Lamiaceae family, encompasses a series of species used on an industrial scale and with a well-described and developed culture process. Extracts of this genus are traditionally used as foods and are highly valued due to the presence of significant amounts of antioxidant phenolic compounds. Many essential oil chemotypes show distinct aromatic flavor conferred by different terpene proportions. Mint extracts and their derived essential oils exert notable effects against a broad spectrum of bacteria, fungi or yeasts, tested both *in vitro* or in various food matrices. Their chemical compositions are well-known, which suggest and even prompt their safe use. In this review, genus *Mentha* plant cultivation, phytochemical analysis and even antimicrobial activity are carefully described. Also, in consideration of its natural origin, antioxidant and antimicrobial properties, a special emphasis was given to mint-derived products as an interesting alternative to artificial preservatives towards establishing a wide range of applications for shelf-life extension of food ingredients and even foodstuffs. *Mentha* cultivation techniques markedly influence its phytochemical composition. Both extracts and essential oils display a broad spectrum of activity, closely related to its phytochemical composition. Therefore, industrial implementation of genus *Mentha* depends on its efficacy, safety and neutral taste.

## 1. Introduction

Medicinal plants represent a significant source of therapeutic remedies, being also the basis of traditional or indigenous healing systems, still widely used by the majority of populations in many countries [1,2]. Recently, the ethnopharmacological potentials of these plant matrices have received important consideration by both scientists and the pharmaceutical industry towards complementing or even replacing conventional pharmacotherapies [2,3,4,5,6,7,8,9,10,11]. Moreover, many of these plants have also been highlighted for their added-food value ability, providing a dual role, i.e., food flavor and bioactive compounds [3,12,13,14,15,16,17,18,19].

*Mentha* species belong to the family Lamiaceae and are widely distributed in Europe, Asia, Africa, Australia, and North America [20,21]. Plants from this genus can be found in multiple and diverse environments [20,22]. Recent data, based on morphological, cytological and genetic characteristics, have shown that genus *Mentha* can be classified into 42 species, 15 hybrids and hundreds of subspecies, varieties and cultivars. Indeed, mint taxonomy is highly complex and there is not always a consensus. The *Mentha* genus is often divided into 5 sections: *Audibertia*, *Eriodontes*, *Mentha*, *Preslia*, and *Pulegium* [23]. Most *Mentha* species are perennial, contain essential oils, and are widely cultivated as industrial crops for essential oil production. A clear definition of an essential oil is mostly based on pharmaceutical standards. Typically, they are odorous products, with a complex composition, obtained from a botanically defined raw plant material by steam or dry distillation, or even by another suitable mechanical process without heating. The term “essential oil” comes from the sense that it contains “the essence of” the plant’s fragrance, not to be confused with its being essential for the functioning of plant cells or plant homeostasis. The main *Mentha* oil producers are USA, India and China [24]. The most economically important species are *Mentha aquatica* L., *Mentha canadensis* L., *Mentha spicata* L. (spearmint) and their hybrids (including *Mentha* × *piperita* L., widely known as peppermint, a sterile and first-generation hybrid between *M. aquatica* and *M. spicata*) [23]. Leaves, flowers and stems of *Mentha* species have traditionally been used as herbal teas and spices in many foods to add aroma and flavor [25,26]. Fresh and dried plant material, raw extracts and essential oils of mint plants are used as a part of confectionary, as flavor enhancing agents in toothpastes, chewing gums and beverages, bakery, cosmetics, as oral hygiene products, pharmaceuticals and pesticides [27]. The two most popular mint flavors are due to (−)-menthol, a terpene alcohol with strong, intense and refreshing peppermint flavor, mostly obtained from *M. aquatica* and *M. canadensis* hybrids, and *R*-(-)-carvone, a terpene ketone with a typical flavor of Maghrebi mint tea or mint cocktails, obtained from *M. spicata*. Another distinct flavor can be obtained from linalool-rich species, a terpene alcohol, for example *M. arvensis* L. Also, (−)-menthol, in its pure form (extracted or synthetized), can also be used in the food and cosmetic industries. Indeed, mint species have a long history of use, and are well-known for their medicinal properties [28]. Mint species, in particular their essential oils, and even menthol have been described in distinct monographs, such as European, Japanese and Russian pharmacopoeias.

*Mentha* leaves have traditionally been used as tea in the treatment of headache, fever, digestive disorders and various minor ailments [26]. Furthermore, mint essential oils have been widely used in the treatment of mild-intensity fungal and bacterial infections of human skin, headache syndromes and postherpetic neuralgia [27]. In modern medicine, *Mentha* species are widely used in the treatment of gastrointestinal tract disorders. For example, *Mentha longifolia* (L.) methanolic extract, rich in eucalyptol, showed antiulcer activity against acetic acid-induced colitis in rats, perhaps attributed to its antioxidant and anti-inflammatory effects, although the observed effect was not dose-dependent [29]. The anti-inflammatory effect of mint essential oils is being corroborated by some clinical studies; for example, *M*. *spicata* essential oil is able to reduce pain in osteoarthritis patients. This analgesic effect is mostly related to the main components of *M*. *spicata* essential oil, like carvone, limonene and menthol [30,31].

The interest in studying mint for human beings is majorly related to its phytosanitary effects. In fact, *M. pulegium* L. essential oil showed high efficiency against the food pest *Sitophilus granaries*, and could additionally be used as a bio-insecticide [32]. *M.* × *piperita* L. aqueous extract improved radish seedlings (*Raphanus sativus* L.) defensive responses against oxidative stress and had inhibitory effects on seed germination and seedling growth, thus representing a huge area of interest for natural herbicide formulation [33]. Mint phenols have also shown strong antioxidant, acetylcholinesterase (AChE), butyrylcholinesterase (BChE) and histone deacetylase inhibitory effects [34,35,36,37,38].

## 2. Genus *Mentha* Plant Cultivation

Mints are a group of perennial herbs commercially cultivated in different parts of the world. It is believed that the genus originated in the Mediterranean basin and, from there, spread to the rest of the world by both natural and artificial means. *M. arvensis* is cultivated on a huge scale in Brazil, China, Paraguay, Japan, Thailand, Angola and India. Peppermint is grown in the USA, Morocco, Argentina, France, Hungary, Italy and Switzerland [39]. Spearmint (*M. spicata*) is mainly cultivated in the USA, and bergamot mint (several hybrids rich in linalool and linalyl acetate) is native to Europe and has been naturalized in the eastern USA. Spearmint and bergamot mint are significantly less cultivated than peppermint. Currently, most of the commercially important mints are hybrids or amphiploids.

### 2.1. Agro-Climatic Requirements

*M. arvensis* can be cultivated both in tropical and sub-tropical areas. It does not grow well in areas with damp winters which cause root rot. A temperature of about 20–25 °C promotes vegetative growth, but essential oil and menthol concentrations have been reported to increase at a higher temperature (30 °C). Peppermint and spearmint cannot be profitably grown in tropical and subtropical areas. Peppermint is grown in cool to temperate regions. It needs long days with warm to hot conditions and cool nights to ensure the right balance of essential oil compounds, produced during the growing phase. Optimum temperature is between 21 and 26 °C for peppermint growth and flowering [40]. Bergamot mint can be grown both in temperate and sub-tropical areas. However, higher yields are observed in temperate climates, and rainfall should be around 100–110 cm. Light showers at planting time and sunny days at the harvesting stage are best for high yield and good quality of leaves.

### 2.2. Soil

Mints are not fussy about soil types and can tolerate a fairly wide range of soil chemistry and conditions. Loam and sandy loam to deep soil, rich in humus is ideal for mint cultivation [41]. The most important factors to consider are pH (6–7.5), organic content, overall water holding capacity and drain ability. Indeed, fresh weight, dry weight and essential oil yield of peppermint are influenced by soil pH [42].

### 2.3. Land Preparation

Land must be cleared with ploughing to release weeds. Mint needs thorough two cross harrowing to bring the soil to fine tilth. About 25–30 tons of farm yard manure (compost)/ha has to be applied as a basal dressing during the last ploughing to incorporate manure with soil. Green manure, like sun-hemp (*Crotalaria juncea* L.), should be used before plantation. Raised beds are the best and most economical way to grow mint.

### 2.4. Propagation

Mints are perennial herbs propagated mainly by vegetative means rather than seeds. In fact, seed propagation has been practiced by some farmers, namely *M. arvensis*, *M. pulegium* and *M. spicata*, as these species produce viable seeds [43]. Peppermint is a popular and commercially grown hybrid mint, but is sterile, therefore its propagation exclusively depends on vegetative parts, like green shoots, underground stolons and rooty turions [44]. El-Keltawi and Croteau [45] have observed a maximum rooting percentage using etiolated rhizome, which was used as a single node cutting. It has also been estimated that 1 ha of well-grown plant material can supply a cropping area of 7 to 10 ha [46].

### 2.5. Planting

Planting materials are cut into small pieces and planted in shallow furrows about 7 to 10 cm deep with a row to row distance of 45–60 cm either manually or mechanically [47]. The plot needs irrigation immediately after planting, and depending on cultivation procedure, soil and climatic factors, plantation lasts about 3 to 5 years.

### 2.6. Fertilization

Exhaustive studies have been made to understand the role of nitrogen (N), phosphorus (P) and potassium (K) requirements on herbage production and essential oil yield. Indeed, N application at 160 kg N/ha for *M. arvensis*, 125 kg N/ha for *M.* × *piperita*, 100–120 kg N/ha for *M. aquatica* and *M. spicata* give a higher dry matter amount and essential oil yield [48,49,50]. Helsel and Fluck [51] have shown a correlation between N fertilizer application and (−)-carvone and limonene concentrations in *Mentha* × *gentilis* L. Generally, 80–120 kg N fertilizer, 50 kg P_2_O_5_ and 40 kg K_2_O/ha is required for a good mint crop. In fact, experiments conducted at the Central Institute of Medicinal and Aromatic Plants (CIMAP, Lucknow, India) have shown that the application of 20 kg/ha sulphur (S) increase *M. spicata* foliage and essential oil yield [46].

### 2.7. Irrigation

Mint requires frequent and adequate irrigation. Fully developed plants are watered at least three times a week. It is important to keep the soil constantly moist, although well drained. The crop has high water demands in summer. Care has to be taken to prevent a waterlogged soil, especially in winter, as this will influence growth [52].

Several studies have shown that peppermint and spearmint biomass production is highly sensitive to water stress [49,53,54,55,56]. Therefore, to increase peppermint yield, furrow irrigation is preferred to sprinkler irrigation, which damages leaf oil cells gland and reduces marketable oil yield [57]. Mitchell and Yang [54] reported that alternate furrow irrigation method is more advantageous over conventional furrow irrigation, as it allows frequent application of small water amounts.

### 2.8. Effect of Cultivation Conditions on Essential Oil Content

Environmental stress can negatively affect the quality of peppermint essential oil. Depending on the edaphoclimatic conditions, the content of phenolics and terpenes can be different. For example, the concentration of menthol in peppermint is higher in flowering stage than during budding period [58]. Research on biotechnological methods for mint oil production has gained pivotal attention due to its economic prospects. Several strategies were attempted for cloning and characterizing key genes involved in mint oil components biosynthesis [59]. DNA library creation encoding oil gland secretory cells and the collection of sequence tag expression provide information on gene expression and metabolic regulation of essential oil biosynthesis [60].

### 2.9. Pests and Diseases

Like other cultivated medicinal plants, mints are also susceptible to exposure to a wide variety of pests and diseases, causing significant crop loss and deterioration of mint oils quality [61]. Different causal organisms, including fungi [61,62,63,64,65,66,67,68], insects [46], nematodes [69,70], bacteria [71] virus and phytoplasma [72,73], are among to the common threats on mint cultivation. Among these fungi, *Puccinia menthae* (rust), *Alternaria alternata* (leaf spot); *Verticillium dahliae* (wilt), *Phoma stasserti* (stem rot), *Rhizoctonia solani* (root and stolon rot) and *Erysiphe cichoracearum* (powdery mildew) cause serious economic loss worldwide. In fact, diseases control through chemicals appears to be more attractive, though alternative strategies like botanicals and biopesticides are also practiced to prevent mint diseases [61,64,74]. Additionally, mint diseases control can also be achieved through breeding programs that develop resistant clones [75].

### 2.10. Harvesting

Mint harvesting must take place at the proper time, since harvesting timings that are too early or too late result in immature or over-mature crops, which ultimately gives poor essential oil quality and quantity [76]. Another important factor is the number of harvests per year, which also influences forage yield and essential oil composition [77]. The crop must be harvested when plants are in full bloom, in the late morning on a dry sunny day, when all dew traces have disappeared. According to climatic conditions, one or two crop flushes are harvested. In addition, mint essential oil quality differs between first and second harvest, as does the oil content between the first and second year of *M. arvensis* harvest [78]. In brief, the plant gives the best crop in second and third year after planting [79,80].

## 3. Phytochemical Composition of Essential Oils Obtained from Genus *Mentha*

### 3.1. Volatile Compounds

Essential oils are usually detached from aqueous phase through a physical process that does not significantly affect their composition. Characteristically appearing in their liquid, volatile, limpid and rarely colored form, essential oils also display a good solubility in lipids and organic solvents, often having lower densities than that of water [81]. Odorous secondary metabolite biosynthesis in *Mentha* species occurs in peltate glandular trichomes, specialized epidermal tissues located on leaves, stems, petals and seed coat surfaces, depending on the species [59,60].

**Figure 1:**
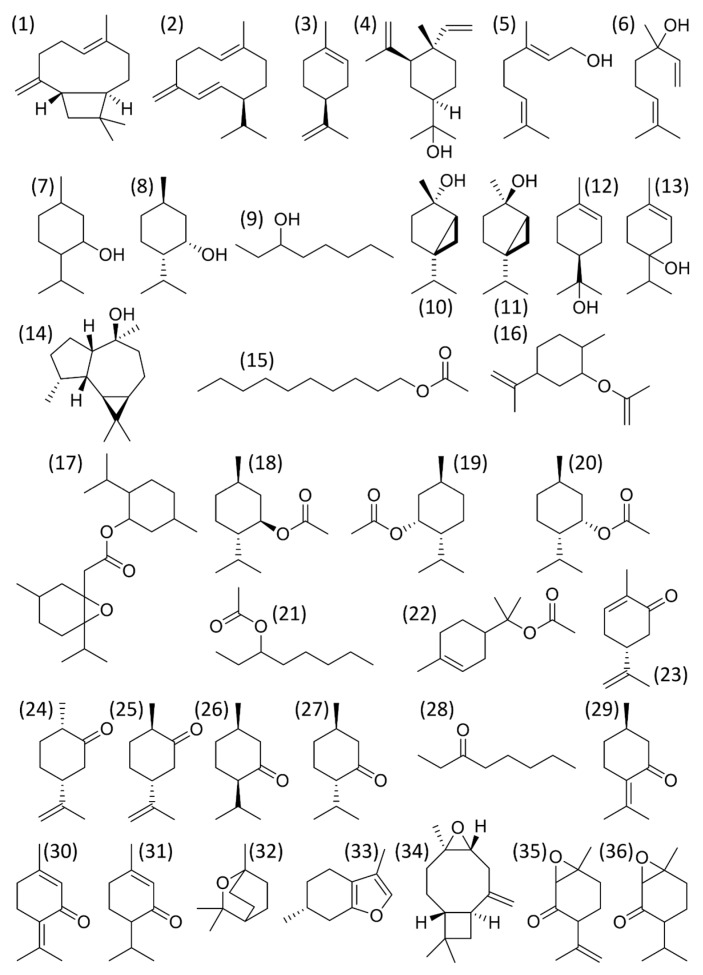
Main components of *Mentha* species essential oils: (1) β-caryophyllene, (2) germacrene D, (3) limonene, (4) elemol, (5) geraniol, (6) linalool, (7) menthol, (8) neomenthol, (9) 3-octanol, (10) cis-sabinene hydrate, (11) trans-sabinene hydrate, (12) α-terpineol, (13) terpinen-4-ol, (14) viridoflorol, (15) decyl acetate, (16) dihydrocarvyl acetate, (17) 1,2-epoxyneomenthyl acetate, (18) menthyl acetate, (19) neoisomenthyl acetate, (20) neomenthyl acetate, (21) 3-octyl acetate, (22) α-terpinyl acetate, (23) carvone, (24) cis-dihydrocarvone, (25) trans-dihydrocarvone, (26) isomenthone, (27) menthone, (28) 3-octanone, (29) pulegone, (30) piperitenone, (31) piperitone, (32) 1,8-cineole, (33) menthofuran, (34) caryophyllene oxide, (35) piperitenone oxide, and (36) piperitone oxide.

Mint plants either produce C3-oxygenated *p*-menthane (e.g., pulegone, menthone, menthol) or C6-oxygenated *p*-menthane (e.g., carvone) types, as major monoterpenes [82]. Hydrocarbons (*β*-caryophyllene, germacrene D, limonene), alcohols (elemol, geraniol, linalool, menthol, neomenthol, 3-octanol, *cis*/*trans*-sabinene hydrate, *α*-terpineol, terpinen-4-ol, viridoflorol), esters (decyl acetate, dihydrocarvyl acetate, 1,2-epoxyneomenthyl acetate, menthyl acetate, neoisomenthyl acetate, neomenthyl acetate, 3-octyl acetate, *α*-terpinyl acetate), ketones (carvone, *cis*-/*trans*-*d*ihydrocarvone, isomenthone, menthone, 3-octanone, pulegone, piperitenone, piperitone), and ethers (1,8-cineole, menthofuran, caryophyllene oxide, piperitenone oxide, piperitone oxide) were also found as main components of *Mentha* species essential oils (Figure 1) [83].

Members of the genus *Mentha* show a great variability in chemical composition, both intra- and inter-species. Reports on chemical composition of various *Mentha* species showed that many factors interfere with essential oil composition, among them environmental (growth location, soil characteristics, moisture presence, temperature, etc.), phenological (phase of the plant collection), plant part used for essential oil extraction (flowers, stems, leaves, entire aerial parts or inflorescences), type of material (fresh or dry), and even methods used for essential oil analysis (Table 1).

Some *Mentha* species present numerous chemotypes, and the distinct chemical patterns are a consequence of their specific metabolic pathways, e.g., linalool, menthol or (−)-carvone pathway [84], and of their intraspecific chemical differences, e.g., chemotypes [85]. So far, the highest number of chemical constituents (*n* = 82) was reported to *M. longifolia* collected in Tajikistan [86]. On the other hand, only three compounds have been identified in essential oil isolated from *M. spicata* leaves grown in Brazil [87]. Indeed, such data markedly depend on the sensitivity of the analytical method used, besides handling, harvesting time and growth conditions.

There have been numerous reports on *M. aquatica* essential oil composition, in which menthofuran was the main compound identified in the largest number of oils analyzed, ranging from 11–70.5% of the total oil content [88,89,90,91,92,93,94,95]. Menthofuran is followed by 1,8–cineole, β-caryophyllene, limonene and viridiflorol, recorded as the most dominant compounds in *M. aquatica* essential oil. It has been reported that *M. aquatica* possess 26 essential oil chemotypes isolated from aerial parts, leaves, inflorescences, seeds or stolons [95]. In addition to the aforementioned constituents, linalyl acetate, piperitenone oxide, pulegone, menthone, elemol, caryophyllene oxide, α-terpinene, isopinocamphone, linalool, menthol, menthyl acetate, germacrene D, dihydrocarveil acetate, menthyl esters and α-pinene are also found in *M. aquatica* essential oil [95].

On the other hand, *M. arvensis* essential oil was found to contain a lower number of chemical constituents (from 7 to 26) than the other members of *Mentha* genus. In the study carried out by Hussain et al. [135], menthol and isomenthone were the most abundant compounds.

Studies on *M. canadensis* essential oil, collected in China, Brazil and India and extracted using hydrodistillation, identified menthol as the main constituent [96,98,99]. Still, in the study performed in China, it was observed that salt stress can reduce menthol concentration at the same time increase menthone and pulegone contents in *M. canadensis* essential oil.

Regarding *M. cervina* essential oil, pulegone and isomenthone were the main constituents identified in all studies performed [85,101,102]. Rodrigues et al. [85] found that cultivated populations belonging to the same chemotype were characterized by the presence of pulegone as the most abundant constituent. Therefore, the studied essential oils contained in their chemical composition oxygenated monoterpenes, with pulegone being the main compound (52–75%), followed by isomenthone, limonene and menthone. These findings allowed authors to classify the obtained oils into two categories: (1) pulegone-rich with low isomenthone content; and (2) pulegone- and isomenthone-rich oils.

Chowdhury et al. [136] investigated the essential oil chemical profile of different *M. spicata* varieties from Bangladesh, and found (−)-carvone and limonene as major constituents, with these data having been recently confirmed by Dwivedy et al. [100]. For *Mentha diemenica* essential oil, collected in Queensland (Australia), menthone, neomenthyl acetate and pulegone were found to be the main compounds present, while essential oil of the same species from Canada had significantly higher amounts of pulegone, clearly supporting it as the main compound, followed by menthone and isomenthone, with neomenthyl acetate not being found [103].

There are a number of studies dealing with the chemical composition of *M. longifolia* essential oils from different geographical origins, showing a great degree of chemical variation, which is not surprising, bearing in mind the very high morphological diversity of this species, resulting in a high number (*n* = 276) of subspecies, varieties and forms. The main constituents of *M. longifolia* essential oil belong to oxygenated monoterpenes group, which include pulegone [108], piperitenone oxide [107] and 1,8-cineole [105]. Beyond these, carvone, isomenthone, borneol, menthol, menthone, piperitenone, dihydrocarvone, limonene, sabinene, α-pinene, eucalyptol, γ-terpineol, β-caryophyllene, isopulegone, cadinene and β-pinene were also recorded as meaningful compounds from *M. longifolia* essential oil.

Indeed, chemical profiling allows the differentiation of many chemotypes, each of them being dominated by one terpene, like piperotenone oxide, carvone, piperitone epoxide, menthone, pulegone, piperitone, *trans*-dihydrocarvone, isomenthone, menthofuran, menthol, 1,8-cineole, isopiperitenone, piperitenone and borneol [86,137,138,139,140,141,142].

For *M. spicata* subsp. *condensata* (Briq.) Greuter and Burdet there is only one study assessing their chemical composition, in which pulegone, piperitenone oxide and piperitenone were identified as the most abundant ones [123].

*M. pulegium* (pennyroyal) essential oil has been extensively studied [109,110,111,112,113,115]. Pulegone was the most abundant compound identified in all the studied essential oils. Other compounds present in relatively high amounts were menthone, piperitenone, piperitone, isomenthone and limonene. In a similar way, *M. spicata* (spearmint) essential oils also have several compounds present as main constituents, corresponding to up to 94.8% of the total oil content [87]. *M. spicata* chemotypes are characterized by (−)-carvone (40.8 to 67.8%) [96,108,113,115,116,118,119,120,122], piperitone oxide (35.7 to 94.8%) [87,121], piperitone (81.2%) [117] or pulegone (53.9%) [96]. So far, series of *M. suaveolens* (grapefruit mint) essential oils chemotypes has been described, with predominance of pulegone [125], (−)-carvone [118,124], linalool [126], piperitone, piperitone oxide, menthone/isomenthone, pulegone/menthone/isomenthone, and pulegone/piperitone [111,118,143]. Oumzil et al. [125] analyzed *M. suaveolens* (grapefruit mint) essential oils collected in various regions of Morocco and concluded that three chemotypes can be defined: pulegone-rich, piperitenone oxide-rich or containing similar proportions of piperitenone and piperitone oxides.

*Mentha* × *rotundifolia* (L.) Huds. is a species originating from *M. longifolia* and *M. suaveolens* hybridization*.* Twenty-three compounds were identified by Lorenzo et al. [112] in the essential oil of an Uruguayan population, where the main constituent (80.8%) was piperitenone oxide.

Considering *M. × piperita* essential oil, studies showed a very high variability derived from the existence of numerous chemotypes. Barrosa et al. [96] studied two different *M. × piperita* varieties (chocolate mint and grapefruit mint varieties) and identified menthofuran, menthone *d*-neoisomenthol and pulegone as the main compounds in a chocolate mint variety, while linalyl acetate with linalool were those prevailing in a grapefruit mint variety. A commercial essential oil purchased in New Delhi (India) presented, in its chemical composition, menthol (19.1%), isomenthone (14.8%) and limonene (10.6%) as major components [127]. *M. × piperita* essential oil collected in Serbia presented a similar composition to that collected in Morocco, with menthol, menthyl acetate and menthone as the main constituents [120,128]. Menthol was also found to be the main compound present in *M. × piperita* essential oils isolated from plant material collected in Iran, China, Taiwan, Saudi Arabia and Brazil [129,130,131,132,133]. However, beyond menthol (up to 59.7%), high amounts of menthone, isomenthone, menthyl acetate and menthofuran were also reported. Another chemotype identified from this species is characterized by its high amount of (−)-carvone, accompanied by limonene as the second main constituent [134].

### 3.2. Non-Volatile Compounds

A wide range of other chemical constituents, mostly phenolic compounds, are also present in mint tissues [144], as briefly shown in Table 2. Interestingly, it should be highlighted that rosmarinic acid, luteolin-7-*O*-glucoside, salvianolic acid, eriocitrin and hesperidin have been found to be the major non-volatile constituents in *Mentha* species [145].

## 4. Food Preservative Applications of Genus *Mentha* Essential Oils

Food manufacturers, regulatory agencies and, finally, consumers have been increasingly concerned about microbiological food safety and the growing number of foodborne illnesses caused by pathogens [146]. According to the World Health Organization’s (WHO) global estimates of foodborne diseases, 600 million people fall ill after consuming contaminated food, of which 420,000 die. In European countries alone, more than 23 million people fall ill every year from unsafe food, and this results in 5,000 deaths [147]. Over 90% of all cases of food poisoning are caused by *Bacillus cereus*, *Campylobacter jejuni*, *Clostridium botulinum*, *Clostridium perfringens*, *Escherichia coli* pathogenic strains, *Listeria monocytogenes*, *Salmonella* spp., *Shigella* spp., *Staphylococcus aureus*, *Yersinia enterocolitica*, as well as *Vibrio cholerae* [148]. These microorganisms are responsible for foodborne outbreaks in different food branches, such as drinking water, beverages, dairy products, fruits, vegetables and even meat and fish products [149]. For decades, in the food industry, a wide variety of synthetic compounds has been used for preservative and antimicrobial purposes with the aim of inhibiting microorganism growth and spoiling. Sodium benzoate, sodium and calcium propionate, sorbic acid, ethyl formate, and sulfur dioxide are examples of chemical substances commercially used to inhibit the growth of microorganisms in foods [150]. Butylated hydroxyanisole (BHA), butylated hydroxytoluene (BHT), tertiary butylated hydroquinone (TBHQ), propyl gallate, ascorbic acid (vitamin C) and tocopherols (vitamin E) are antioxidants used as food preservatives [150]. However, it has also been stated that the widespread use of synthetic preservatives has led to huge ecological and medical problems, which, in addition to economic considerations, have triggered the search for new/safer strategies against microbiota spoilage [151,152]. Due to the growing consumer demand for safe, high-quality and healthy food with reduced quantity of synthetic preservatives or antimicrobials, an increasing interest has been stated on natural antimicrobials from plants [153]. It has long been known that plant phytochemicals protect against viruses, bacteria, fungi and herbivores, but it has only recently been learned that they can be also used for food spoilage microorganism protection.

One of the most popular and representative plant groups is the *Lamiaceae* family. Nowadays, it is used both in traditional and modern medicine, as well as in the pharmaceutical and food industries. The use of mint is not strictly limited to essential oils, which are widely recognized for their strong aromatic properties. Indeed, essential oils and their derived extracts can be effectively used as natural food preservatives. As a result, they can fulfill several important tasks: prolong shelf-life, eliminate synthetic preservatives and food flavors, as well as forming part of a healthy food trend that influences market sales. Due to the overall popularity and occurrence, mint appear to be relatively well tested in terms of antibacterial activity against a wide spectrum of bacteria, such as *E. coli* pathogenic strains, *L. monocytogenes*, *Salmonella* spp., *S. aureus*, and many others (Table 3). Curiously, the vast majority of studies on *Mentha* spp. antimicrobial effects have been linked to essential oils and plant extracts.

### 4.1. In vitro Studies on Mentha Genus

#### 4.1.1. Extracts

Methanolic extracts from six *Mentha* species (*M. aquatica*, *M. arvensis*, *M. × piperita*, *M. pulegium*, *M. × rotundifolia* and *M. × villosa* Huds.) exhibited a powerful antioxidant activity, ranging from 7.5 to 44.7 μg/mL, which supports their upcoming use as natural food preservatives [154]. For example, *M. × piperita* extract, rich in flavonoids, at a concentration of 5 mg/mL reduced the *in vitro* growth of two cereal fungi, *Phoma sorghina* and *Fusarium moniliforme* around 72% and 55%, respectively; dimethylsulfoxide (DMSO) at 1% DMSO was added to the culture media as a control [155]. Sujana et al. [156] reported that *M. × piperita* leaf extract is more active against *S. aureus*, *Bacilus subtilis* and *Proteus vulgaris* than *E. coli* (positive control: chloramphenicol 100 μg/mL). Bayoub et al. [157] reported that the minimal inhibitory concentration (MIC) of *M. suaveolens* ethanol extract against *Listeria monocytogenes* subsp. Timija was significantly lower (0.3 mg/mL) than MIC obtained for cinnamon (0.4 mg/mL), cistus (0.5 mg/mL), rose (0.9 mg/mL), thyme (1.6 mg/mL), wild thyme (2.2 mg/mL), artemisia (3.8 mg/mL), rosemary (5.3 mg/mL), geranium (6.2 mg/mL), chamomile (6.8 mg/mL), lavender (11.5 mg/mL) and verbena (11.8 mg/mL) (positive controls: penicillin G 10 units, nalidixic acid 30 μg, vancomycin 30 μg, tetracycline 30 μg, chloramphenicol 30 μg, novobiocin 5 μg, and ampicillin 5 and 30 μg). Moreover, Bupesh et al. [158] found prominent antipathogenic effects of peppermint leaf water extract against *B. subtilis*, *Pseudomonas aeruginosa*, *Staphylococcus aureus* and *Serratia marcescens* by an *in vitro* agar well diffusion method (positive control: chloramphenicol 100 μg/mL). Dhiman et al. [159] tested the effect of different extracts from *M. arvensis* prepared using different solvents (acetone, methanol, ethanol, water) against microorganisms isolated from spoiled juices: *B. cereus*, *Serratia* spp., *Rhodotorula mucilaginosa*, *Aspergillus flavus* and *Penicillium citrinum*. The obtained results showed that ethanol extract activity was similar to that of sodium benzoate (100 mg/mL), a commonly used preservative in beverage production. Beyond direct antimicrobial activity, *M. × piperita* essential oils and extracts also inhibited *L. monocytogenes*, *P. aeruginosa*, *Asaia lannensis*, *Asaia bogorensis* and *Candida* species *(C. albicans* and *C. dubliniensis)* biofilm formation and development [160,161]. The final concentration of the extracts in the wells was 1 mg/mL, while ciprofloxacin at a concentration of 2,5 μg/mL and amphotericin B at 5 μg/mL were used as the positive controls for *P. aeruginosa* and *C. albicans*, respectively [160]; for the other microorganisms, the control samples were the same culture media but without *M. × piperita* [161]. Panda et al. [162] showed that *M. arvensis* aqueous extract at 0.8 mg/mL inhibited citrinin production by *P. citrinum* up to 73% (positive control: none).

#### 4.1.2. Essential Oils

Essential oils derived from different mint species have shown significant antibacterial activity against human pathogenic microorganisms, such as *S. aureus*, *Micrococcus flavus*, *B. subtilis*, *Staphylococcus epidermidis* and *Salmonella enteritidis* [133]. *M. × piperita* essential oil, with (−)-carvone (35%), pulegone (15%), methyl petroselinate (16%) as main compounds, demonstrated antimicrobial activity against Gram-positive (*S. aureus*) and Gram-negative (*E. coli*) bacteria [134]. Essential oils from *M. aquatica*, *M. longifolia* or *M. × piperita* aerial parts were markedly active against pathogenic fungi, like *C. albicans*, *Epidermophyton floccosum*, *Microsporum canis* and *Trichophyton* spp. *(T. mentagrophytes*, *T. rubrum* and *T. tonsurans)* [138,177]. *M. × piperita* essential oil showed significant antifungal activity against *A. alternata*, *Fusarium tabacinum*, *Penicillum* spp., *Fusarium oxyporum* and *Aspergillus fumigatus* [133]. Studies conducted by Mahboubi and Haghi [178] reported that *M. pulegium* essential oil exhibited antimicrobial activity against *S. aureus*, *L. monocytogenes*, *B. cereus*, *E. coli* and yeast *C. albicans.* The data obtained by the authors pointed out that *M. pulegium* essential oil activity was comparable to well-known antibiotics vancomycin, erythromycin or gentamycin. In a similar way, Ait-Ouazzou et al. [179] highlighted that *M. pulegium* essential oil antibacterial activity against *S. aureus*, *L. monocytogenes*, *S. enteritidis* and *E. coli* was higher than galingale (*Cyperus longus*) and juniper (*Juniperus phoenicea*)*.* Dhifi et al. [180] also reported that *M. spicata* essential oil showed high activity against Gram-positive *Staphylococcus (S. epidermidis* and *S. aureus)* and Gram-negative *Salmonella* and *E. coli* species. Soković et al. [181] found that *M. × piperita* and *M. spicata* essential oils were more active against pathogenic bacteria *B. subtilis*, *E. coli*, *Pseudomonas* (*P. mirabilis*, *P. aeruginosa*), *Salmonella* (*S. enteritidis*, *S. typhimurium)* and *S. aureus* spp. than essential oils from sweet basil (*Ocimum basilicum* L.), lavender (*Lavandula angustifolia* Mill.), bitter orange (*Citrus × aurantium* L.), sage (*Salvia officinalis* L.) and chamomile (*Matricaria chamomilla* L.)*.* The authors also noted that menthol was more active than linalyl acetate, limonene, β-pinene, α-pinene, camphor, linalool and 1,8-cineole.

On the other hand, Ben Arfa et al. [182] compared the antimicrobial activity of carvacrol with that of carvacrol methyl ether, carvacrol acetate, eugenol, and menthol. Menthol showed very weak antibacterial activity, with it being suggested that the benzene ring is of huge importance to promoting strong effects. Indeed, menthol molecules have a cyclohexane ring, which seems to make this compound less active. Nonetheless, compounds present in essential oils and extracts show multiple actions and even synergistic effects. Consequently, mixtures can be more effective as food preservatives than pure substances. The main mechanisms of antimicrobial synergy include: (1) sequential steps inhibition in specific biochemical pathways; (2) inhibition of enzymes that degrade of excrete antimicrobial agents; and (3) cell wall/membrane interaction leading to increased uptake of other antimicrobials [183]. For example, in yeasts, the results obtained by Ferreira et al. [184] indicated that *M. × piperita* essential oil was able to induce cell death in *Saccharomyces cerevisiae* due to pro-oxidant effects, both in cytosol and mitochondria.

Synergy can not only be observed between different constituents of one essential oil or extract but also in a mixture of them. For example, the combination of *Lippia multiflora* Moldenke and *M. × piperita* essential oils showed synergetic effects against *E. coli*, *Enterococcus faecalis*, *Enterobacter aerogenes*, *L. monocytogenes*, *P. aeruginosa*, *Shigella dysenteriae*, *S. aureus* and *Salmonella* (*S. enterica* and *S. typhimurium)* species [185]. Soković et al. [181] and Riahi et al. [172] indicated that, in general, essential oils show stronger action against Gram-positive than Gram-negative bacteria. In fact, it has been shown that the lower susceptibility of Gram-negative bacteria results from the presence of hydrophobic lipopolysaccharide in their outer cell membrane, which confers protection against different active agents [170]. This bacterial structure prevents depolarization and pore formation, while at the same time increasing membrane permeability or fluidifying effect [183].

There are some crucial factors affecting antimicrobial activity, including essential oil composition, active substances concentration and type of microorganisms tested. First of all, essential oil composition may be determined by some plant cultivation factors, such as environmental conditions, harvesting period, drying method, storage conditions as well as extraction methods [186]. Although the different mint varieties from which plant extracts and essential oils are derived are characterized by strong and multifaceted *in vitro* activities against bacteria, yeasts and molds, it should be clearly emphasized that their biological activity may vary considerably in industrial conditions for specific food matrices.

Table 4 lists *in vitro Mentha* species activities against fungi and yeasts*.* Results from *in vitro* studies for *M. × piperita* essential oils against several *Aspergillus (A. flavus*, *A. fumigatus*, *A. oryzae*, *A. clavatus)*, *Candida* (*C. albicans*, *C. glabrata*, *C. tropicalis*, *C. krusei*, *C. dubliniensis*, *C. parapsilosis)* and *Cryptococcus neoformans* species showed prominent effects at relatively low concentrations, ranging from 0.5 to 4 μL/mL [187]. According Pandey et al. [188], *M. arvensis* essential oil displayed a high toxicity on *Penicillium italicum* than lemon grass (*Cymbopogon citratus*) and sweet basil (*O. basilicum*) essential oils. *Mentha* spp. essential oils used for *Aspergillus parasiticus* control inhibited aflatoxin synthesis [189]. In the work performed by Kumar et al. [190], *M. arvensis* essential oil was tested against 9 postharvest fungi and exhibited absolute inhibition of *Aspergillus* species (*A. flavus*, *A. fumigatus*, *A. ochraceus*, *A. niger*, *A. terreus)*, *Helminthosporium oryzae* and *Sclerotium rolfsii*. Still, essential oil significantly exhibited growth in all tested fungi at a concentration of 500 ppm, with the observed activity being similar to basil (*Ocimum americanum* L.) essential oil [191]. Soković et al. [120] investigated *M. spicata*, *M. × piperita* and thymes (*Thymus vulgaris* L. and *T. sibthorpii* Benth.) activity against 17 plant, animal and human pathogens. Results showed that *M. spicata* essential oil had a greater fungistatic activity than *M. × piperita*, but a weaker one than *T. vulgaris*. In addition, the authors found that all tested essential oils showed stronger antimicrobial activities than bifonazole against *A. alternata*, *Aspergillus* (*A. niger*, *A. ochraceus*, *A. versicolor*, *A. flavus*, *A. terreus)*, *Cladosporium cladosporioides*, *Fusarium tricinctum*, *Penicillium (P. ochrochloron*, *P. funiculosum)*, *Phomopsis helianthi*, *Trichoderma viride*, and *Trichophyton* (*T. mentagrophytes*, *T. rubrum*, *T. tonsurans)* species.

## 5. Shelf-Life Prolongation

Despite *Mentha* spp. plant extracts and essential oils having shown great *in vitro* antimicrobial effects against bacteria, yeasts and molds, their action can be quite different in complex environments, such as food matrices. Indeed, essential oils’ biological activity is markedly influenced by food components (e.g., fats, carbohydrates, proteins, water, salt, preservatives), temperature, pH, water activity and packaging methods [193]. The negative impact of high protein content on essential oil activity was reported by Tassou et al. [163], who studied the effect of mint essential oils on *S. enteritidis* and *S. aureus* growth. The authors attributed antimicrobial activity reduction to protein content, suggesting that the phenolic group monoterpenes could bind to proteins, thereby lowering the number of antimicrobial compounds available. Cava et al. [194] tested the antimicrobial activity of mint essential oils against *L. monocytogenes* in milk, and found that essential oils bioactivity was reduced by fats. Diverse studies reported that essential oils exhibited highest activity at low pH levels [195,196]. However, other parameters also affect essential oil activity, like temperature and sodium chloride. Combination of carvacrol and *p*-cymene with sodium chloride (1.3 g/L) showed an antagonistic effect. On the other hand, it is well known that high sodium chloride concentration contributes to cell lysis. Going further, essential oils show better antimicrobial activity in vapor than in liquid phase [197].

Both extracts and essential oils derived from different mint species may extend food products shelf-life (Table 5).

*M. longifolia* leaf extract (6.0%) application shows better antioxidant and antimicrobial activities than cumin (*Cuminum cyminum* L.) seed extract. Wild mint extracts significantly affected fresh rainbow trout shelf-life, extending it by up to 12–18 days during storage in a refrigerator, which suggests that they can be effectively applied as natural preservatives in fish products shelf-life extension. Sensory analysis showed that rainbow trout treatment with *M. longifolia* extract improved overall quality and sensory properties [204].

Results obtained by Tassou et al. [210] showed that *M. × piperita* essential oil at concentrations ranging from 0.5 to 2.0% completely reduced *S. enteritidis* number in tzatziki, and markedly decreased its number in taramosalata. In the same study, *L. monocytogenes* populations showed a declining trend in a one-week storage period. However, this effect was not observed for pâté. The authors speculated that mint essential oils’ antibacterial action depends not only on essential oil concentration, but also on food product (type, pH, storage temperature) and type of spoilage microbiota. Klūga et al. [198], reported that *M. × piperita* leaf extract protected fish from spoilage. Also, mint extract treatment suppressed total *Enterobacteriaceae* and *Pseudomonas* spp. bacteria growth in mackerel. Moreover, in these food matrices, mint extract inhibited lipid oxidation, and then enhanced stability storage and extended shelf life by 2 to 5 days [201]. Nonetheless, mint essential oils and extracts can also be successfully used in other food products. The study of Choi et al. [199] demonstrated peppermint oil antibacterial activities against *Acidovorax citrulli*, a bacteria responsible for watermelon blotch. These results suggested the possibility of using peppermint oil as an antibacterial agent to treat contaminated seeds. In addition to that, a mint-supplemented cereal biscuit (in different forms) enriched in natural antioxidants, maintained acceptable for consumption over 5 months storage period. Polyphenols’ antioxidant efficiency prevented biscuit rancidity onset during storage; therefore, mint may be conceived of as a key substitute for synthetic antioxidants in baked food product preservation [203].

Finally, essential oil synergy is also an interesting point [31]. *M. × piperita* essential oil and silver ions (Ag^+^) combination acted synergistically against *E. coli*, *S. aureus* and *C. albicans* cultures [211]. Also, *M. × piperita* essential oil (0.5%) and bacteriocin (1000 AU/g) combination delayed microorganism spoilage proliferation in stored minced beef meat. Thus, biopreservative effect in combination can be considered a promising tool for upcoming application in meat products preservation [212].

## 6. Conclusions and Future Perspectives

Among Lamiaceae family, the *Mentha* genus encompasses several species already used at industrial scale and with well-developed cultivation. Extracts are traditionally used as food, and contain remarkable antioxidant phenolic compounds. Many essential oil chemotypes show distinct aromatic flavor conferred by different terpenes proportions. Both extracts and essential oils show activities on a broad spectrum of microorganisms tested *in vitro* as well as using various food matrices. Due to its natural origin, antioxidant and antimicrobial activities, mint-derived products could become a great alternative to artificial preservatives, and to find a wide range of applications for shelf-life extension of fruits and vegetables, beverages, dairy products, baking or meat and fish products. Nevertheless, industrial implementation depends on efficacy, ease of use and profitability of such natural additive over synthetic preservatives.

Extracts could be obtained as essential oil by-products, allowing a higher profitability, and where its moderate activity can be compensated by their safety. Besides that, essential oil taste is not neutral, and therefore its wide aroma should be carefully adapted to the matrices used in order to minimize the aromatic impact, and might also be used for food ingredient preservation before processing. Not least important to emphasize is that the finished products could also be marketed with the explicit mention of such natural preservatives to facilitate overall acceptance and demand. Further organoleptic studies should be performed, along with antimicrobial studies to assess the relevance of such ingredient in finished food products.

## Figures and Tables

**Table 1:** Main findings on phytochemical analysis of *Mentha* species.

Plant			Extraction			Compounds		References
Part	Material	Origin	Method	Identification	Yield	Number	Most Abundant	
***Mentha aquatica* L.**								
ns	Fresh	Romania	HD	GC-MS	ns	41	menthofuran (58.59%), limonene (9.91%) trans-β-ocimene (5.59%) ledol (3.29%) β-caryophyllene (3.55%)	[94]
AP	Dried	Vietnam	SD	GC and GC–MS	0.42% (TH) 0.34% (NA)	28	Thanh Hóa province: -epi-bicyclosesquiphellandrene (58.9%) -limonene (21.1%) Nghệ an province: -epi-bicyclosesquiphellandrene (52.4%) -limonene (31.4%)	[95]
AP	ns	Brazil	HD	GC–MS	0.26%	19	d-carvone (58.79%) limonene (28.29%).	[96]
***Mentha arvensis* L.**								
L	Fresh	Brazil	DHM	GC-MS	ns	7	Young leaves: -menthol (81.46%) -pulegone (7.25%) -*p*-cymene (3.27%) Mature leaves: -menthol (86.87%) -*p*-cymene (4.38%) -pulegone (3.47%)	[97]
ns	ns	Brazil	SD	GC–MS	ns	26	menthol (56.85%) isomenthone (21.13%) menthyl acetate (4.62%) limonene (4.07%) isopulegol (3.71%)	[83]
AP	-	Brazil	HD	GC–MS	0.10%	21	linalyl acetate (39.72%) linalool (34.57) 1.8-cineole (10.04%)	[96]
***Mentha canadensis* L.**								
AP	Fresh	China	HD	GC GC–MS	0.02–0.18%	16	menthol (80.47%) menthone (7.25%) isomenthone (2.31%) isopulegol (2.17%) pulegone (2.05)	[98]
AP	ns	China	HD	GC and GC–MS	1.42%	36	menthol (28.8%) α-pinene (16.4%) menthone (12.7%) α-terpineol (6.3%) limonene (5.5%)	[99]
AP	ns	Brazil	HD	GC–MS	0.54%	24	menthol (46.98%) isomenthone (29.07%)	[96]
***Mentha cardiaca* J. Gerard ex Baker**								
AP	ns	India	HD	GC–MS	12 mL/kg	60	carvone (59.6%) limonene (23.3%) β-myrcene (2.5%) 1,8-cineole (2.1%) *cis*-dihydrocarvone (1.5%) β-Bourbonene (1.5%)	[100]
***Mentha cervina* L.**								
AP	Dried	Portugal	HD	GC GC–MS	ns	28	August (flowering phase): -pulegone (75.1%), isomenthone (8.7%) -limonene (4.3%) Vegetative phases: -October: pulegone 79.6%, isomenthone 9.6%, limonene (3.2%) -December: pulegone 58.3%, isomenthone 33.3%, limonene 1.2% -February: pulegone 12.9%, isomenthone 77.0%, menthon 4.4%	[101]
AP	ns	Portugal	HD	GC GC–MS	ns	33	pulegone (52–75%) isomenthone (8–24%) limonene (4–6%) menthone (1–2%)	[102]
AP	ns	Portugal	HD	GC GC–MS	2.4–4.0%	25	cultivated populations: pulegone (62–78%) isomenthone (3.1–15%) limonene (3.4–7.4%) wild populations. pulegone (73–80%) isomenthone (6.1–18.2%) limonene (4.5–5.2%)	[85]
***Mentha diemenica* Spreng.**								
L	ns	Canada Australia	SD	GC GC–MS	1.0%	35	Australia: -menthone (32.4%) -pulegone (24.9%) -neomenthyl acetate (18.3%) -neomenthol (9.0%) -menthyl acetate (5.7%) -menthol (1.8%) -isomenthone (1.0%) Canada: -pulegone (43.6%) -menthone (32.2%) -isomenthone (3.2%) -menthyl acetate (2.7%) -neomenthol (2.5%) -menthol (2.7%)	[103]
***Mentha longifolia* L.**								
L	Air-dried	Turkey	HD	GC-MS	ns	40	menhone (19.31%) pulegone (12.42%) piperitone (11.05%) dihydrocarvon (8.32%) limonene (6.1%) 3-terpinolenone (5.66%) 1,8-cineole (4.37%) germacrene D (3.38%) caryopyllene (3.19%)	[104]
L	Dried	Iran	HD	GC-MS	1.34%	24	Leaf oil: -1,8-cineole (37.16%) -piperitenone oxide (18.97%) -sabinene (13.94%) -α-pinene (8.92%) -pulegone (6.14%).	[105]
S	Dried	Iran	HD	GC-MS	0.76%	27	Stem oil: -1,8-cineole (36.81%) -pulegone (18.61%) -piperitenone oxide (12.21%) -sabinene (7.05%).	
F	Dried	Iran	HD	GC-MS	0.97%	25	Flower oil: -piperitenone oxide (37.67%) -1,8-cineole (23.02%) -sabinene (13.56%) -α-pinene (10.45%)	
L	Fresh	Saudi Arabia	HD	GC-MS	0.5–0.9%.	49	pulegone (11.92–62.54%) menthone (7.84–34.13%) eucalyptol (5.96–20.07%)	[106]
AP	Dried	Bosnia and Herzegovina	HD	GC-MS	1.9%	36	piperitone oxide (63.58%) 1.8-cineole (12.03%) caryophyllene oxide (4.33%) *trans*-caryophyllene (2.98%) *cis*-caryophyllene (0.82%)	[107]
AP	Dried	Tajikistan	HD	GC-MS	0.5–0.9%.	82	*cis*-piperitone epoxide (7.8–77.6%) piperitenone oxide (1.5–49.1%) carvone (0.0–21.5%) menthone (0.0–16.6%) thymol (1.5–4.2%) pulegone (0.3–5.4%) β-thujone (0.2–3.2%) (*E*)-caryophyllene (0.9–2.5%) myrcene (0.3–2.5%) carvacrol (0.0–2.7%) borneol (0.9–1.8%) *p*-cymene (0.2–1.9%)	[86]
L	Fresh	Tunisia	HD	GC-MS	1.3%	35	pulegone (54.41%) isomenthone (12.02%) 1,8-cineole (7.41%) borneol (6.85%) piperitenone oxide (3.19%)	[108]
AP	ns	Brazil	HD	GC–MS	0.05%	11	piperitenone oxide (60.79%) l-limonene (13.81%) carvone (5.21%) myrcene (2.51%) β-pinene (1.92)	[96]
***Mentha pulegium* L.**								
AP	Dried	Algeria	HD	GC GC-MS	2.34%	37	pulegone (46.31%) piperitenone (23.3%) menthone (6.2%) limonene (4.7%)	[109]
AP	Dried	Morocco	HD	GC-MS	5.4%	21	pulegone (40.98%) menthone (21.164%) humulene (5.4%) eucalyptol (5.2%)	[110]
AP	Dried	Morocco	HD	GC GC-MS	ns	10	pulegone (73.33%) menthone (8.63%) α-pinene (1.70%)	[111]
AP	Dried	Uruguay	HD	GC GC-MS	1.93%	22	pulegone (73.4%) isomenthone (12.9%) menthone (3.6%)	[112]
AP	Dried	Algeria	HD	GC GC-MS	1.0%	43	pulegone (38.81%) menthone (19.24%) piperitenone (16.52%) piperitone (6.34%) isomenthone (6.09%) limonene (4.29%) octanol (1.85%)	[113]
AP	Dried	Morocco	HD	GC-MS	5.4%	21	pulegone (40.98%) menthone (21.164%)	[110]
AP	Dried	Portugal	HD	GC-MS	0.90%	53	menthone (35.9%) pulegone (23.2%) neo-menthol (9.2%)	[114]
L	Dried	Algeria	SD	GC-MS	1.45%	39	pulegone (70.66%) neo-menthol (11.21%) menthone (2.63%) *cis*-isopulegone (2.33%) piperitenone 1.58%	[32]
AP	Dried	Morocco	SD	GC GC-MS	2.0%	29	pulegone (73.0%) isomentone (13.4%) menthone (2.6%) limonene (1.4%)	[115]
***Mentha spicata* L.**								
L	Fresh	India	HD	GC GC-MS	6.5 mL/kg	18	carvone (48.60%) *cis*-carveol (21.30%) limonene (11.30%)	[116]
L	ns	Brazil	HD	GC-MS		25	piperitone (81.18%) piperitenone (14.57%) limonene (1.47%)	[117]
AP	Dried	Tunisia	HD	GC/MS		34	carvone (40.8%) limonene (20.8%), 1,8-cineole (17.0%) β-pinene (2.2%) *cis*-dihydrocarvone (1.9%) dihydrocarveol (1.7%).	[118]
L	Fresh	Tunisia	HD	GC-MS	0.8%	49	carvone (50.47%) 1,8-cineole (9.14%) limonene (4.87%) camphor (3.68%) β-caryophyllene (3.0%)	[108]
AP	Dried	Algeria	HD	GC GC-MS	0.87%	57	carvone (59.40%) limonene (6.12%) 1,8-cineole (3.80%) germacrene D (4.66%) β-caryophyllene (2.96%) β-bourbonene (2.79%) α-terpineol (1.98%) terpinene-4-ol (1.12%)	[113]
AP	Fresh	Senegal	HD	GC GC-MS	0.10%	30	carvone (67.8%) limonene (18.1%) *cis*-dihydrocarvone (1.9%) *trans*-carveol (2.9%) (*E*)-β-caryophyllene (1.1%) germacrene D (1.1%)	[119]
AP	Dried	Senegal	HD	GC GC-MS	0.19%	34	carvone (74.7%) limonene (12.5%) *cis*-dihydrocarvone (2.0%) *trans*-carveol (2.2%) (*E*)-β-caryophyllene (1.1%) germacrene D (1.0%)	[119]
AP	Dried	Serbia	HD	GC-MS	ns	27	carvone (49.5%) menthone (21.9%) limonene (5.8%) 1,8-cineole (3.0%)	[120]
AP	Fresh	Brazil	SD	GC	0.32%	3	piperitone oxide (94.8%) γ-muurolene (1.06%) β-farnesene (0.76%)	[87]
AP	Dried	Greece	HD	GC GC-MS	0.2%	39	piperitenone oxide (35.7%) 1,8-cineole (14.5%) trans-calamene (6.4%) spathulenol (5.2%)	[121]
AP	Fresh	India	HD	GC/FID GC-MS	0.56%	20	carvone (49.62 to 76.65%) limonene (9.57 to 22.31%) 1,8-cineole (1.32 to 2.62%) trans-carveol (0.3 to 1.52%)	[122]
AP	Dried	Morocco	SD	GC GC-MS	0.70%	43	carvone (42.3%) limonene (11.0%) menthone (7.2%)	[115]
***Mentha spicata* subsp. *condensata* (Briq.) Greuter & Burdet**								
AP	Fresh	Italy	HD	GC GC-MS	0.21%	29	pulegone (34.1%) piperitenone oxide (32.9%) piperitenone (11.3%) (*Z*)-ocimene (3.9%) limonene (3.9%) linalool (2.7%)	[123]
***Mentha suaveolens* Ehrh.**								
AP	Dried	Morocco	HD	GC GC-MS	ns	15	piperitenone (33.03%) pulegone (17.61%) piperitone (9.18%)	[111]
F	Fresh	Egypt	HD	GC GC-MS	1.7%	29	carvone (50.59%) limonene (31.25%) *trans*-β-caryophyllene (2.56%)	[84]
AP	Fresh	Egypt	HD	GC-MS	0.47–0.60%	46	Spring, summer and autumn samples: -carvone (31–56%) -limonene (22.59–29.18%) Winter samples: -limonene (26%) -carvone (25%)	[124]
AP		Morocco	SD	IR, NMR and MS	0.012%	31	pulegone (0.1–50%) piperitenone oxide (0.9–56%) piperitone oxide (0.3–26%)	[125]
L	Dried	China	HD	GC-MS	1.08%	28	linalool (41.50%) linalyl anthranilate (33.75%) α-terpineol (6.29%) geranyl acetate (3.67%) nerol acetate (2.09%) *trans*-geraniol (2.07%)	[126]
AP	Dried	Tunisia	HD	GC-EIMS	1.1%	34	carvone (40.8%) limonene (20.8%) 1,8-cineole (17.0%)	[118]
***Mentha × rotundifolia* (L.) Huds.**								
AP	Dried	Uruguay	HD	GC GC-MS	1.02%	23	piperitenone oxide (80.8%) (Z)-sabinene hydrate (2.0%) 4-terpineol (1.5%)	[112]
***Mentha × piperita* L.**								
AP	Fresh	Senegal	HD	GC GC-MS			menthofuran (30.7%) pulegone (17.6%) 1.8-cineole (3.7%) menthol (15.9%) menthone (13.0%)	[119]
AP	Dried	Senegal	HD	GC GC-MS			menthofuran (28.1%) pulegone (13.8%) 1.8-cineole (3.4%) menthol (15.9%) menthone (14.2%)	[119]
AP	Dried	Serbia	HD	GC-MS	ns	26	menthol (37.4%) menthyl acetate (17.4%) menthone (12.7%)	[120]
ns	ns	India	HD	Oil: GC and GC-MS Oil vapours: SPME GC-MS		Oil: 47 Vapour: 18	Oil: -menthol (19.1%) -isomenthone (14.8%) -limonene (10.6%) -isomenthanol (8.8%) -menthyl acetate (6.6%) -β-pinene (5.6%) Oil vapour: -α-pinene (17.3%) -limonene (18.4%) -β-pinene (13.9%) -isomenthone (9%) -menthyl acetate (6.6%) -β-phellandrene (5.8%)	[127]
L	Dried	Morocco	HD	GC GC-MS	1.02%	29	menthone (29.01%) menthol (5.58%) menthyl acetate (3.34%) menthofuran (3.01%) 1,8-cineole (2.40%)	[128]
L	Dried	Iran	HD	GC GC-MS	1.38%	35	menthone (30.63%) menthol (25.16%) menthofuran (6.47%) β-phellandrene (5.59%) isomenthone (4.74%) menthol acetate (4.61%) pulegone (4.39%) β-caryophyllene (3.05%) neomenthol (2.83%) 1,8-cineole (2.15%)	[129]
AP	Dried	Iran	HD HS/SPME	GC GC-MS	0.42%	HD: 39 HS/SPME: 41	HD oil: -menthol (45.34%) -menthone (16.04%) -menthofuran (8.91%) HS/SPME oil: -menthol (29.38%) -menthone (16.88%) -cis-carane (14.39%) -menthofuran (11.38%) -1,8-cineole (9.45%)	[130]
L	ns	China	HD	GC-MS	ns	51	menthol (30.69%) menthone (14.51%) menthyl acetate (12.86%) neomenthol (9.26%) pulegone (4.36%) cineole (2.91%) caryophyllene (2.52%)	[131]
AP	Fresh	Taiwan	HD	GC-MS	0.3%	10	menthol (30.35%) menthone (21.12%) *trans*-carane (10.99%) isomenthol (6.26%)	[132]
AP	Dried	Saudi Arabia	HD	GC-MS	ns	19	menthol (36.02%) menthone (24.56%) menthyl acetate (8.95%) menthofuran (6.88%)	[133]
ns	ns	Brazil	SD	GC-MS	ns	36	menthol (59.73%) isomenthone (18.45%) methyl acetate (6.02%) neomenthol (2.43%) isopulegol (2.15%)	[83]
L	Dried	Oman	HD	GC–MS	ns	14	carvone (34.94%) pulegone (14.89%) methyl petroselinate (15.51%) d-limonene (11.20%) p-cineole (5.70%)	[134]
AP	Dried	Morocco	SD	GC GC-MS	1.40%	37	linalool (41.4%) linalyl acetate (39.5%)	[115]
AP	Dried	Brazil	HD	GC-MS	0.10%	18	d-carvone (49.27%) limonene (37.18%)	[96]
AP	Fresh	Senegal	HD	GC	0.28%	29	linalool (45.8%) linalyl acetate (42.7%) 1.8-cineole (2.0%)	[119]
AP	Dried	Senegal	HD	GC-MS	0.21%	34	linalool (42.0%) linalyl acetate (38.5%) 1.8-cineole (3.1%)	[119]

AP, all plant; DHM, dichloromethane; EIMS, electron impact mass spectrometry; F, flowers; FID, flame ionization detector; GC, gas chromatography; HD, hydrodistillation; HS/SPME, headspace/solid-phase micro-extraction; IR, infrared spectroscopy; L, leaves; MS, mass spectrometry; NMR, nuclear magnetic resonance; ns, not specified; S, seeds; SD, steam distillation; SPME, solid phase micro extraction.

**Table 2:** Major non-volatile compounds in *Mentha* species.

Chemical Constituents	Individual Compounds
*Anthocyanidins*	Cyanidin, delphinidin, luteolinidin, pelargonidin, petunidin
*Coumarins*	Esculetin and scopoletin
*Flavanols*	Catechin, epicatechin
*Flavanones*	Eriocitrin, eriodictyol, hesperidin, naringenin, narirutin
*Flavones*	Apigenin, diosmetin, diosmin, luteolin, luteolin-*O*-glucuronide, gardenin B, luteolin-*O*-glucoside, pebrellin, salvigenin, thymusin, thymonin
*Flavonols and dihydroflavonols*	Quercetin, kaempferol, rutin
*Phenolic acids*	Cinnamic acid, its analogs (hydroxybenzoic, *p*-coumaric, ferulic, caffeic, sinapic, rosmarinic, salvianolic, isosalvianolic, didehydrosalvianolic and lithospermic acids, nepetoidin A/B) and glycosides (caffeic acid glucuronide, chlorogenic, caftaric), gallic, syringic and vanillic acids
*Phenylethanoids*	Tyrosol
*Stilbenoids*	Resveratrol
*Terpenes*	Oleanolic acid

**Table 3:** *Mentha* spp. activity against bacterial pathogens tested *in vitro*.

Plant Species	Bacterial Strain	References
***Mentha × piperita* L.**	Gram negative: Proteobacteria *Escherichia coli* *Klebsiella pneumoniae* *Proteus mirabilis*, *P. vulgaris* *Pseudomonas aeruginosa* *Salmonella enteritidis*, *S. paratyphi* A and B, *S. pullorum*, *S. typhi*, *S. typhimurium* *Shigella dysenteriae* *Yersinia enterocolitica* Gram positive: Firmicutes *Bacillus cereus*, *B. subtilis* *Listeria monocytogenes* *Staphylococcus aureus* *Streptococcus pyogenes*	[156,158,163,164,165,166,167,168,169]
***Mentha suaveolens* L.**	Gram negative: Proteobacteria *Escherichia coli* *Klebsiella pneumoniae* *Pseudomonas aeruginosa* *Proteus mirabilis* Gram positive: Firmicutes * Bacillus anthracis* *Staphylococcus aureus*	[125]
***Mentha spicata* L.**	Gram negative: Proteobacteria *Escherichia coli* *Klebsiella pneumoniae* *Proteus mirabilis* *Pseudomonas aeruginosa* *Salmonella typhimurium* *Vibrio* spp. Gram positive: Firmicutes *Bacillus cereus*, *B. subtilis* *Listeria monocytogenes* *Staphylococcus aureus*	[118,170,171]
***Mentha × rotundifolia* (L.) Huds.**	Gram negative: Proteobacteria *Escherichia coli* *Salmonella typhimurium* Gram positive: Firmicutes *Bacillus cereus* *Staphylococcus aureus*	[172]
***Mentha arvensis* L.**	Gram negative: Proteobacteria *Escherichia coli* *Klebsiella pneumoniae* *Pseudomonas aeruginosa* *Shigella flexneri* Gram positive: Firmicutes *Staphylococcus aureus*	[173]
***Mentha longifolia* L.**	Gram negative: Proteobacteria *Escherichia coli* *Pseudomonas aeruginosa* *Salmonella typhimurium* Gram positive: Firmicutes *Bacillus cereus* *Listeria monocytogenes* *Staphylococcus aureus* *Streptococcus pyogenes*	[171,174]
***Mentha pulegium* L.**	Gram negative: Proteobacteria *Escherichia coli* *Pseudomonas aeruginosa* *Salmonella typhimurium* Gram positive: Firmicutes *Bacillus cereus* *Staphylococcus aureus*	[175,176]

**Table 4:** *Mentha* spp. activity against fungi and yeasts tested *in vitro.*

**Plant Species**	Yeast/Fungi Strain	References
***Mentha arvensis* L.**	*Penicillium citrinum*	[162]
***Mentha longifolia* L.**	*Candida albicans*	[174]
***Mentha × piperita* L.**	*Candida albicans*	[172]
	*Aspergillus flavus* *Aspergillus parasiticus* *Fusarium solani* *Sclerotium rolfsii* *Candida albicans*	[168]
***Mentha × rotundifolia* (L.) Huds.**	*Candida albicans*	[172]
***Mentha suaveolens* L.**	*Candida albicans*	[125]
***Mentha × piperita* essential oils in chitosan–cinnamic acid nanogel**	*Aspergillus flavus*	[192]

**Table 5:** *Mentha* spp. essential oil or extract application and food shelf-life prolongation.

Plant Species	Spoiling Microorganisms	Food Matrix	Reference
***Mentha × piperita* L.**	Bacteria Gram negative: Proteobacteria *Acinetobacter pittii* *Acinetobacter baumannii* *Buttiauxella agrestis* *Delftia acidovorans* *Enterobacter cloacae* *Escherichia coli* *Lelliottia amnigena* *Pantoea agglomerans* *Pseudomonas alcaligenes* *Pseudomonas oryzihabitans* *Providencia rettgeri* *Rahnella aquatilis* *Serratia liquefaciens* Gram positive: Firmicutes *Staphylococcus caprae* *Staphylococcus epidermidis*	Fish	[198]
***Mentha × piperita* L.**	Bacteria Gram negative: Proteobacteria *Acidovorax citrulli* (bacterial fruit blotch)	Watermelon	[199]
***Mentha × piperita* L.**	Bacteria Gram negative: Proteobacteria *Escherichia coli*	Commercial chicken soup	[200]
***Mentha arvensis* L. with citrus peel extract**	Aerobic plate count	Mackerel	[201]
***Mentha spicata* L.** ***Mentha pulegium* L.**	Fungi *Debaryomyces hansenii*	Doogh	[202]
***Mentha spicata* L.**	Antioxidant properties	Biscuits	[203]
***Mentha longifolia* L.**	viable aerobic bacteria, psychrotrophic bacteria	Rainbow trout (fish)	[204]
***Mentha pulegium* L.**	Bacteria Gram positive: Firmicutes *Listeria monocytogenes*	White cheese	[205]
**Mint essential oil combined with HPP process**	Bacteria Gram positive: Firmicutes *Listeria monocytogenes* *Listeria innocua*	Yogurt drink (ayran)	[206]
**Mint extract**	Total count	Tomato juice	[207]
	Bacteria Gram negative: Proteobacteria *Escherichia coli* *Staphylococcus typhimurium*	Fresh-cut lettuce and purslane	[208]
**Mint powder**	Standard plate count Yeast/mould count	Chicken slices	[209]
